# The frequency of somatic mutations in cancer predicts the phenotypic relevance of germline mutations

**DOI:** 10.3389/fgene.2022.1045301

**Published:** 2023-01-09

**Authors:** Edoardo Luigi Draetta, Dejan Lazarević, Paolo Provero, Davide Cittaro

**Affiliations:** ^1^ University of Milan, Milan, Italy; ^2^ Center for Omics Sciences, IRCCS San Raffaele Scientific Institute, Milan, Italy; ^3^ Department of Neurosciences “Rita Levi Montalcini”, University of Turin, Turin, Italy

**Keywords:** mutations, gene prioritization, machine learning, cancer, human disease

## Abstract

Genomic sequence mutations can be pathogenic in both germline and somatic cells. Several authors have observed that often the same genes are involved in cancer when mutated in somatic cells and in genetic diseases when mutated in the germline. Recent advances in high-throughput sequencing techniques have provided us with large databases of both types of mutations, allowing us to investigate this issue in a systematic way. Hence, we applied a machine learning based framework to this problem, comparing multiple models. The models achieved significant predictive power as shown by both cross-validation and their application to recently discovered gene/phenotype associations not used for training. We found that genes characterized by high frequency of somatic mutations in the most common cancers and ancient evolutionary age are most likely to be involved in abnormal phenotypes and diseases. These results suggest that the combination of tolerance for mutations at the cell viability level (measured by the frequency of somatic mutations in cancer) and functional relevance (demonstrated by evolutionary conservation) are the main predictors of disease genes. Our results thus confirm the deep relationship between pathogenic mutations in somatic and germline cells, provide new insight into the common origin of cancer and genetic diseases, and can be used to improve the identification of new disease genes.

## 1 Introduction

Many diseases, both rare and common, are caused or favored by mutations in the germline genome. Even for monogenic diseases, identifying the causative mutation is a difficult problem, since every individual typically carries many loss-of-function mutations that are compatible with a normal phenotype (MacArthur and Tyler-Smith, 2010). Several approaches have been developed to prioritize variants and genes, most of which are based on combining information about the predicted effect of the variants, their frequency in the human genome, and the known or predicted function of the affected gene, as reviewed in (Moreau and Tranchevent, 2012; Eilbeck et al., 2017; Zolotareva and Kleine, 2019).

Cancer is also a disease of the genome, since in most cases it is initiated by mutations occurring in somatic cells, leading to uncontrolled proliferation and eventually to metastatic invasion of other tissues. It is thus natural to ask to what extent the same mutations can be associated to cancer and genetic diseases when occurring respectively in somatic or germline cells. Indeed many cases are known of genes involved in both types of diseases: for example rasopathies (Rauen, 2013) are a family of developmental diseases caused by germline mutations in genes of the Ras/MAPK pathway, which is also recurrently mutated in many cancer types (Stephen et al., 2014). This observation extends to other cancer related genes, such as ATRX and KDM5C involved in X-linked mental disability (Gibbons et al., 1995), KMT2D and KDM6A in Kabuki syndrome (Ng et al., 2010; Lederer et al., 2012), SQSTM1 in Paget disease (Ralston and Albagha, 2014), and DMNT3A in Tatton-Brown-Rahman syndrome (Tatton-Brown et al., 2014), while mutations of chromatin remodelers play crucial roles in both cancer and neurodevelopmental disorders (Ronan et al., 2013). Recently, a significant overlap was found between 285 newly identified genes associated to developmental delay and cancer drivers (Kaplanis et al., 2020), and cancer-driving mutations were found to be enriched in genomic regions intolerant of variation (Vitsios et al., 2022). Here, we set out to investigate systematically the extent to which the mutational spectrum of cancer and that of genetic disorders overlap.

Recent development in sequencing technologies allow the determination of mutations in patients in a fast and cost-effective way–especially when the sequencing is limited to exons–so that sequencing is now routinely used as a diagnostic and prognostic tool in both genetic diseases and cancer. These developments have also allowed the creation of large databases of mutations including many thousands of individuals, providing us with the means to investigate the relationship between somatic and germline pathogenic mutations in a systematic and statistically controlled way.

**FIGURE 1:**
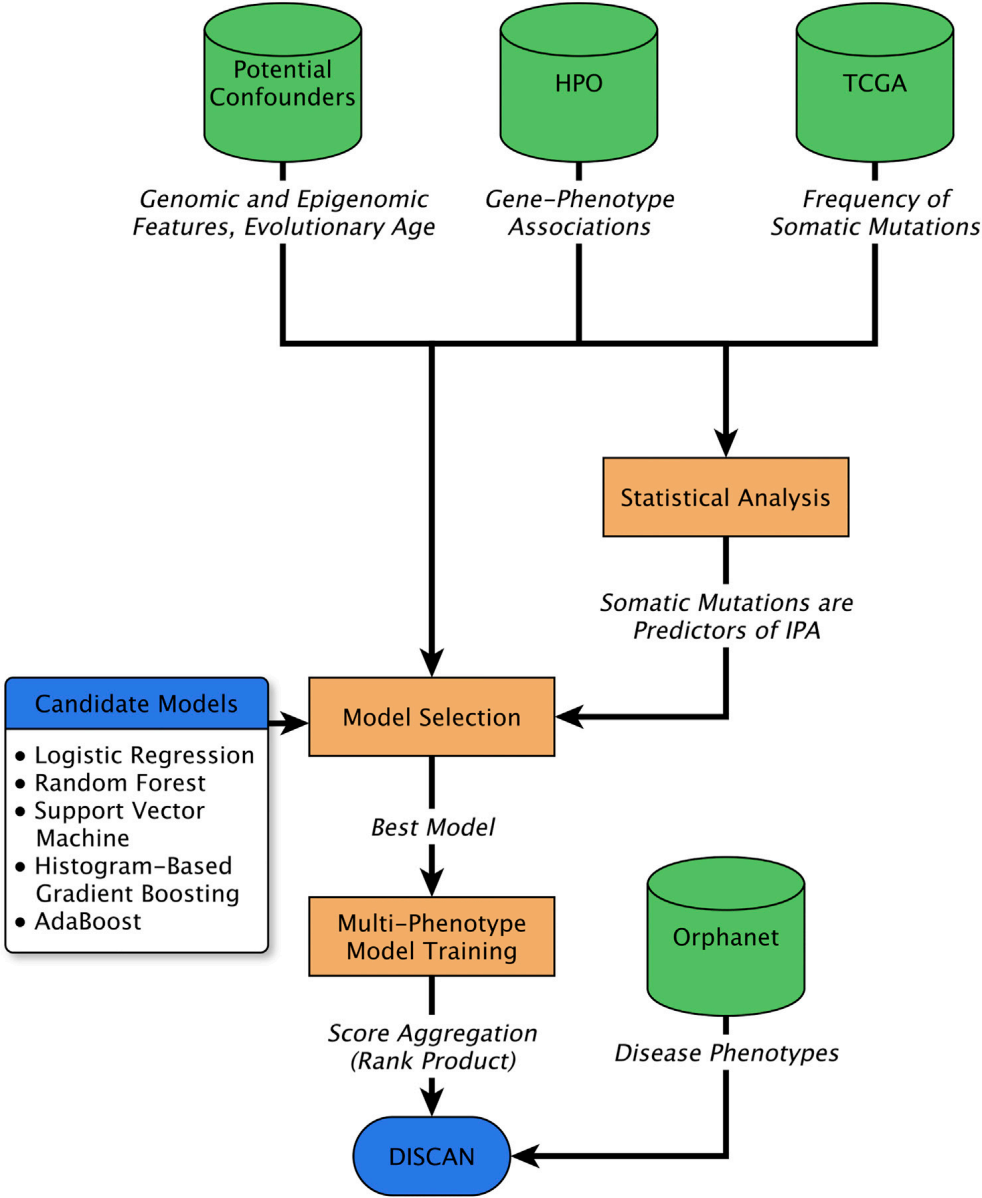
Schematic representation of the procedure adopted in this work. The total number of somatic mutations (TSM) profiled in large cancer datasets (TCGA) were used, together with gene-phenotype associations obtained from the Human Phenotype Ontology (HPO), and several potential confounders of their association, including gene evolutionary age, genomic factors and epigenomic features. Statistical analysis (multivariable logistic regression) was used to establish TSM as significant and independent predictor of genes involved in abnormal phenotypes (IPA). In addition, several machine learning algorithms were trained to predict IPA from the same set of features. The best performing paradigm was chosen and trained to generate independent predictive models for multiple phenotypes. The predictions for phenotypes associated to any disease were aggregated to perform disease gene prioritization.

We thus decided to investigate whether patterns of somatic mutations detected in cancer samples contain information that can be used to predict the involvement of genes in genetic diseases. We chose to tackle the issue in a statistical learning framework, that is to reframe the question as whether it is possible to predict the involvement of a gene in a genetic disease (or, more generally, an abnormal phenotype due to germline mutations) using the frequency of its somatic mutations in a set of common cancers. In this way we can take advantage of statistical methods to accurately quantify the predictive power of the model, and thus the extent of the overlap between the genetics of cancer and of congenital diseases (Figure 1).

## 2 Materials and methods

### 2.1 Gene-phenotype associations

Gene-phenotype associations were obtained from the Human Phenotype Ontology (HPO) (Köhler et al., 2021) web site (release 2020/06/08 for the analysis of “phenotypic abnormality”; 2022/06/11 for the validation analysis, the specific phenotypes, and the disease gene prediction model) together with the ontology graph. We considered a gene associated to a phenotype when it was directly associated to the phenotype itself or to any of its descendants in the HPO. All associations to the HPO term “Neoplasm” (HP:0002664) and to all its descendants were removed before expanding the gene annotations to HPO ancestors. Therefore, for example, a gene associated to “Glioma” was not considered associated to “Abnormal nervous system morphology”, unless the same gene was also associated to another descendant of “Abnormal nervous system morphology”, not descending from “Neoplasm”.

### 2.2 Somatic mutations in cancer

The frequency of cancer somatic mutations of a gene was obtained from the data generated by the Cancer Genome Atlas (TCGA) program, and was defined as the number of patients with one or more somatic mutations in the gene, classified as Frame_Shift_Del, Frame_Shift_Ins, In_Frame_Del, In_Frame_Ins, Missense_Mutation, Non-sense_Mutation, Silent, Splice_Site, or Translation_Start_Site. Protein-changing mutations were defined as those not classified as Silent. Mutation data were obtained from the Firehose repository (https://gdac.broadinstitute.org/).

### 2.3 Gene evolutionary age

The evolutionary age of each gene, expressed as the branch of the tree of life where the gene appeared, was obtained from the Supplementary Material of (Neme and Tautz, 2013) and was used in regression either as a numerical variable assuming integer values between 1 and 20 or as a 20-level categorical variable.

### 2.4 Genomic and epigenomic features

The following genomic and epigenomic features (a total of 81 variables associated to each gene) were computed for each gene and used in the predictive models.

• Coding sequence length (one variable): from UCSC known genes, genome version hg19
• DNAse accessibility (two variables, gene-level and coding sequence [CDS]-level): fraction of the gene region (or the CDS) that overlaps a DNAse I hypersensitive site (DHS) in the “clustered” Encode dataset
• Histone marks (six variables: H3k4me1, H3k4me3, and H3k27ac at the gene and CDS levels): fraction of the gene region (or the CDS) that overlaps a peak of the histone mark. The peaks were obtained for the GM12878, H1-hESC, HSMM, HUVEK, K562, NHEK, and NHLF cell lines from the Encode broad peaks. For each histone mark, peaks from all cell lines were merged.
• Lamin B1 association (one variable): fraction of the gene region overlapping lamin B1-associated domains in Tig3 cells from the corresponding UCSC track
• Replication timing (one variable): replication timing by repli-chip from ENCODE/FSU averaged over the gene region for the BG02ES, GM06990, H1-hESC, H7-hESC, H9ES, HeLaS3, IMR90, and iPS_hFib2_iPS4 cell lines, then averaged over the cell lines
• Nucleosome positioning (two variables, gene-level and CDS-level): nucleosome signal from GM12878 and K562 cells, averaged over the gene (or the CDS) region, then averaged between the 2 cell lines
• Transcription (one variable): pooled RNA-seq signal for the GM12878, H1-hESC, HSMM, HUVEC, K562, NHEK, and NHLF cell lines, averaged over the gene region and then over the cell lines
• Repeat (two variables, gene-level and CDS-level): fraction of the gene region (or the CDS) that overlaps a simple repeat according to the corresponding UCSC track
• Recombination rate (one variable): recombination rate from the corresponding UCSC track averaged over the gene region
• Trinucleotide frequencies (64 variables): frequency of each trinucleotide in the CDS. The CDS sequence was obtained from the UCSC known genes, genome version hg19, in the gene strand

### 2.5 Disease models and genes

Evolutionary age, cancer somatic mutations, and the genomic/epigenomic features could be retrieved for a total of 18,170 protein-coding genes, which were considered in the logistic regression and machine learning analyses. OMIM and Orphanet datasets were retrieved from https://hpo.jax.org/app/download/annotation. Gene Ontology Term Enrichment was performed using g:Profiler (Raudvere et al., 2019), with default parameters.

### 2.6 Logistic regression

We defined the involvement in phenotypic abnormality (IPA) of a gene as the binary variable taking value one if the gene is associated to the HPO term “phenotypic abnormality” (HP:0000118) or any of its ontology descendants, after removal of the neoplasm-associated genes described above. We used univariable and multivariable logistic regression to identify the significant predictors of IPA. All numeric non-binary features were rescaled to zero mean and unit standard deviation prior to fitting the logistic model. For multivariable regression, the 80 genomic/epigenomic features (after the removal of the frequency of the “TTT” trinucleotide which is collinear to the other trinucleotide frequencies) were transformed into their principal components. All principal components were retained in the model.

### 2.7 Machine learning

Six classification paradigms were tested: logistic regression, random forest, support vector machine, AdaBoost, histogram gradient boosting, and Gaussian naïve Bayes. Hyperparameters were tuned using nested cross-validation. Specifically, the dataset was first split into 10 outer-folds, used in turn as test sets. The remaining nine folds were used to tune the hyperparameters through grid search, by dividing them into three inner folds that were used in turn as the validation set. Balancing through random oversampling was performed within the training of the models by cross-validation using the imbalanced-learn package and native scikit-learn functionality. The performance of the model with the optimal hyperparameters was then evaluated by computing the area under the receiver operating characteristic curve (AUROC) on the test set, and the average AUROC across the 10 outer folds was the performance measure. The description of hyperparameter’s space is reported in Supplementary Table S1.

## 3 Results

### 3.1 The frequency of somatic mutations in cancer predicts the involvement in abnormal phenotypes

In order to assess whether the frequency of cancer somatic mutations of a gene is a predictor of its involvement in phenotypic abnormality (IPA), we first retrieved the associations between genes and phenotypes from the Human Phenotype Ontology (HPO) (Köhler et al., 2021). For each (*gene, phenotype*) pair we thus defined a binary variable taking value 1 (0) if the gene is (is not) associated to the phenotype (or to any of its descendants in the ontology). This binary variable was used as the response in the logistic regression and machine learning analyses described below. All cancer-related phenotypes (*i.e.* the HPO term “Neoplasm” and all its descendants) and the related gene associations were excluded from the analysis, since for these phenotypes an overlap between the relevant somatic and germline mutations is expected. Here, we are specifically interested in investigating the relationship between somatic mutations in cancer and non-cancer-related abnormal phenotypes and diseases.

The independent variable of interest is the frequency of somatic mutations of each gene in human tumors. These were obtained from the TCGA research network (https://www.cancer.gov/tcga). For each of the 33 tumor types and each protein-coding gene we extracted the number of patients in which the gene was mutated, thus we associating to each (*gene, tumor*) pair an integer number.

**FIGURE 2:**
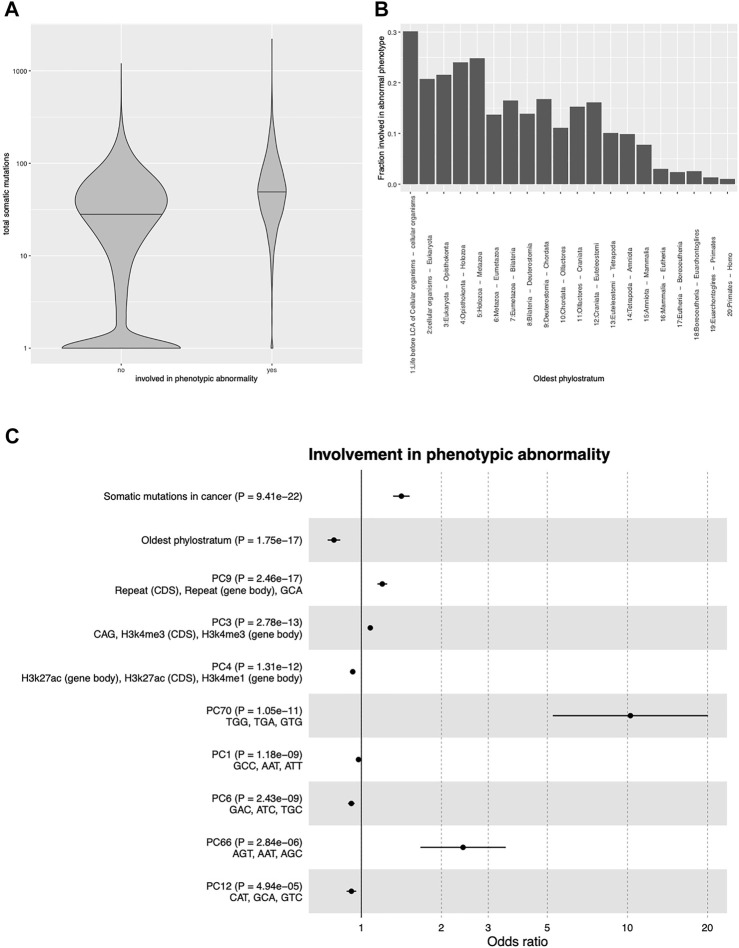
Predictors of involvement in phenotypic abnormality **(A)**: Number of cancer samples with somatic mutations in genes involved or not involved in phenotypic abnormalities according to the Human Phenotype Ontology. **(B)**: Fraction of genes involved in phenotypic abnormalities as a function of gene age. The *x*-axis shows the branching of the tree of life at which the gene appeared according to the reconstruction of Neme and Tautz (2013). **(C)**: Odds ratios and *p*-values of each predictor of involvement in phenotypic abnormality in a multivariable logistic regression with cancer somatic mutations, gene age, and the principal components of 80 genomic/epigenomic features. The top 10 predictors by *p*-value are shown. For each PC, the three top features by loading are shown. CDS, coding sequence; PC, principal component; trinucleotides refer to their respective frequency in the coding sequence.

We first focused on the most general HPO term “phenotypic abnormality”, and considered as the independent variable the number of patients with somatic mutation in each gene, summed over all TCGA tumor types (henceforth denoted by TSM - total somatic mutations). Univariate logistic regression showed that TSM was strongly and positively associated with IPA (OR 1.918, 95% CI 1.851-1.989, *p* < 2.2 ⋅ 10^–16^; the OR is computed in units of one standard deviation). Figure 2A compares the TSM of genes involved vs. not involved in phenotypic abnormalities according to the HPO.

Many factors could in principle confound this association, the simplest example being the length of the gene sequence: Longer genes have higher probability of undergoing mutations, leading to higher probability of being somatically mutated in cancer, but also of undergoing potentially damaging mutations in the germline. More generally, all factors influencing the propensity of a gene to undergo a mutation could explain the association between TSM and IPA. A less obvious confounder is the evolutionary age of the gene, defined as the node in the phylogenetic tree in which the gene first appeared, which can be established for each human gene with phylostratigraphic methods (Neme and Tautz, 2013). It has been shown that evolutionarily older genes are more likely to be involved in genetic diseases (Domazet-Loso and Tautz, 2008) and to carry cancer-relevant mutations (Domazet-Loso and Tautz, 2010). Indeed we confirmed that gene age was strongly and positively correlated with IPA (Figure 2B).

We thus repeated the analysis while correcting for these factors. We used as covariates the evolutionary age of each gene as evaluated in (Neme and Tautz, 2013) and a set of 80 sequence- and epigenetic-related variables (see Methods), including in particular trinucleotide sequence composition, histone modifications, DNA accessibility, recombination rate, and replication timing, all of which have been shown to affect the propensity of a gene to accumulate mutations (see *e.g.* (Stamatoyannopoulos et al., 2009; Michaelson et al., 2012; Schuster-Böckler and Lehner, 2012; Sabarinathan et al., 2016; Halldorsson et al., 2019; Li and Luscombe, 2020)). After transforming the 80 genetic/epigenetic features into their principal components (PCs) to avoid collinearity, we fitted a multivariable logistic regression model to predict IPA from TSM, gene age, and the 80 PCs, including the 18,170 genes for which all variables were available. TSM and gene age were confirmed to be the strongest predictors of disease involvement, and to be independent of each other and of the PCs. The odds ratios and *p*-values of the top 10 predictors are shown in Figure 2C. As the number of positive cases (4,134 genes with IPA) is ∼50 times larger than the number of independent variables (82) used in the logistic model, we do not expect significant overfitting. Indeed the area under the receiver operating characteristic curve (AUROC) of the model was equal to 0.679, while the average AUROC of a 10-fold cross-validated logistic regression was 0.667 (95% CI 0.658-0.676).

This result was robust with respect to several alternative modeling choices (Supplementary Figure S1): (a) considering gene age as a categorical variable instead of a numerical one; (b) using as predictor the rate of patients with a somatic mutation in the gene in each tumor type, averaged over the tumor types (this weighs all tumor types equally irrespective of the number of samples included in the TCGA); (c) considering only somatic mutations predicted to alter the protein sequence.

Importantly, TSM remained a significant and independent predictor of IPA when the status of a gene as cancer driver (derived from (Martincorena et al., 2017) was included as a further covariate (Supplementary Figure S2). Thus the frequency with which a gene is somatically mutated in cancer is associated to IPA independently of whether such mutations contribute to oncogenesis. It should be noted that driver status was the strongest predictor of IPA in this model, in agreement with the overlap between cancer drivers and disease genes found in previous studies (Kaplanis et al., 2020; Vitsios et al., 2022).

### 3.2 A machine-learning predictor of genes associated to abnormal phenotypes

The results of the logistic regression suggest that TSM and gene age, combined with genomic and epigenomic features, could be used to build a predictor of phenotypic relevance. Such a predictor would be, in particular, useful for the prioritization of variants found in the exome sequencing of probands affected by genetic diseases, especially when combined with other prioritization methods based on allele frequencies or pathway annotation.

While the logistic regression approach used above is suitable to assess the statistical significance and independence of the features, machine-learning tools might provide better predictive performance, in particular by bypassing the constraint of linearity. We thus tested, by cross-validation, the performance of several tools in predicting IPA from our features, on the same 18,170 genes used in logistic regression. We eventually selected random forests, which outperformed logistic regression, support vector machines, AdaBoost, histogram gradient boosting, and Gaussian naïve Bayes (see Supplementary Table S2 for the performance comparison and Supplementary Table S1 for model parameters).

**FIGURE 3:**
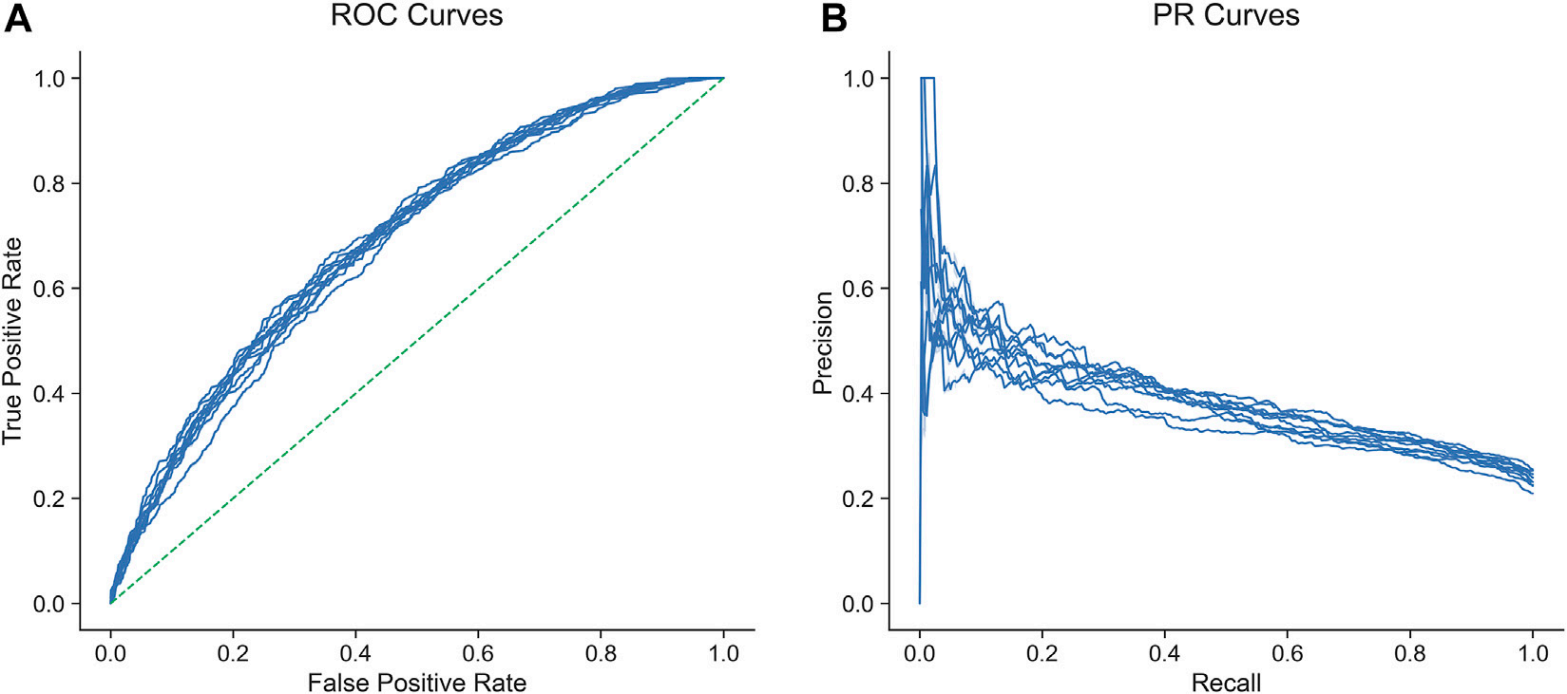
Receiver operating characteristic **(A)** and precision-recall **(B)** curves of ten cross-validated random forest models for the prediction of involvement in phenotypic abnormality from total somatic mutations, gene age, and genomic/epigenomic features.

In predicting IPA from our 83 features, a random forest model achieved an AUROC of 0.688 (95% CI 0.682-0.694) (mean AUROC from ten-fold cross-validation) and an area under the precision-recall curve (AUPRC) of 0.382 (95% CI 0.378-0.397; gain over baseline AUPRC 67%). The ROC and PRC curves of the ten cross-validation folds are shown in Figure 3. Feature importance analysis confirmed that the most predictive features were TSM and gene age (Supplementary Figure S3). TSM and gene age were among the top predictors by importance in all models with the exception of the worst performing one, namely Gaussian naïve Bayes (see Supplementary Table S2).

To determine whether our model was able to predict recently discovered associations between genes and abnormal phenotypes, we took advantage of the fact that a sizable number of genes (435) were associated to abnormal phenotypes between 2020 and 2022, as shown by a comparison of the June 2020 release of the HPO (used for training our models) and the latest release (June 2022). To create a dataset for this test we removed from the 2020 dataset these 435 genes and also 435 randomly chosen genes that were not associated with abnormal phenotypes in either release of the HPO, thus obtaining a balanced test set of 870 genes. The remaining 17,300 were used to train a random forest model. When used on the test set, this model obtained an AUROC of 0.679 (*p* < 2.2 ⋅ 10^–16^), very close to the average AUROC of 0.688 obtained from the cross-validation of the original model. Similar results were obtained by removing a larger fraction of negative cases from the original dataset (1,542 genes) so as to obtain a test set with a fraction of positives similar to the real one (22%). The AUROC in this case was 0.683 (*p* < 2.2 ⋅ 10^–16^). These results show that the model is indeed able to predict new gene-phenotype associations.

These results show that TSM and gene age, combined with genomic/epigenomic features, can be used in a machine-learning framework to build a strongly significant predictor of IPA. The practical use we envision for this predictor is the prioritization of variants found in exome sequencing assays of probands of genetic diseases whose causal mutation is unknown. It could be used in combination with predictors based, for example, on measures of selection based on allele frequencies in human populations, such as (Karczewski et al., 2020); and with those based on functional annotation, such as (Robinson et al., 2014).

However, the predictor described above was trained on genes involved in a generic phenotypic abnormality, while predictors for specific phenotypes would be more useful in the context of variant prioritization. Thus we built a separate random forest model for each HPO term, after discarding the terms with less than 100 associated genes to limit the risk of overfitting, thus training a total of 1,361 models. These models achieved a median cross-validated AUROC of 0.682. The baseline value of the AUPRC (*i.e.*, that expected from a random predictor) is equal to the fraction of positive cases, which varies widely among the phenotypes: Therefore we computed for each phenotype the fractional gain over baseline AUPRC, finding a median gain of 122%.

To show the effectiveness of this approach we took advantage of a recently published catalog of genes linked to Autism Spectrum Disorder (Fu et al., 2022). There, two gene lists are defined with different stringency criteria (ASD72, more stringent, and ASD185, less stringent). For each list we considered genes that were not included in our training set for phenotype “autistic behavior” (HP:0000729) and analyzed the distribution of the rank-transformed probabilities of being associated to the phenotype according to the random forest model (Supplementary Figure S4). We found distributions highly skewed to low ranks (*i.e.* more significant) and significantly different from the uniform distribution of ranks of all remaining genes (*p* = 1.173 ⋅ 10^–12^ for ASD72 and *p* = 1.261 ⋅ 10^–34^ for ASD185, Mann-Whitney test).

These models for specific phenotypes were then used to build predictors for genetic diseases, which are often characterized by multiple phenotypes, as described below.

### 3.3 Combining phenotypes to predict disease genes

Genetic diseases are often characterized by multiple abnormal phenotypes. We reasoned that a promising strategy to predict the causal gene of such a disease would be to combine the predictors of each of the relevant phenotypes. To this end, we collected data from OMIM (Hamosh et al., 2005) and Orphanet (www.orpha.net). In both cases, we extracted the list of unique HPO phenotypes associated to each disease. Phenotypes for which we did not generate a model were mapped to the closest scored node in the HPO ontology. We then rank-transformed the model probabilities ordering these in ascending order, so that the lower the rank value the stronger the gene-phenotype association. Lastly, we computed the disease scores by taking the geometric mean of the ranks of the associated phenotypes.

**FIGURE 4:**
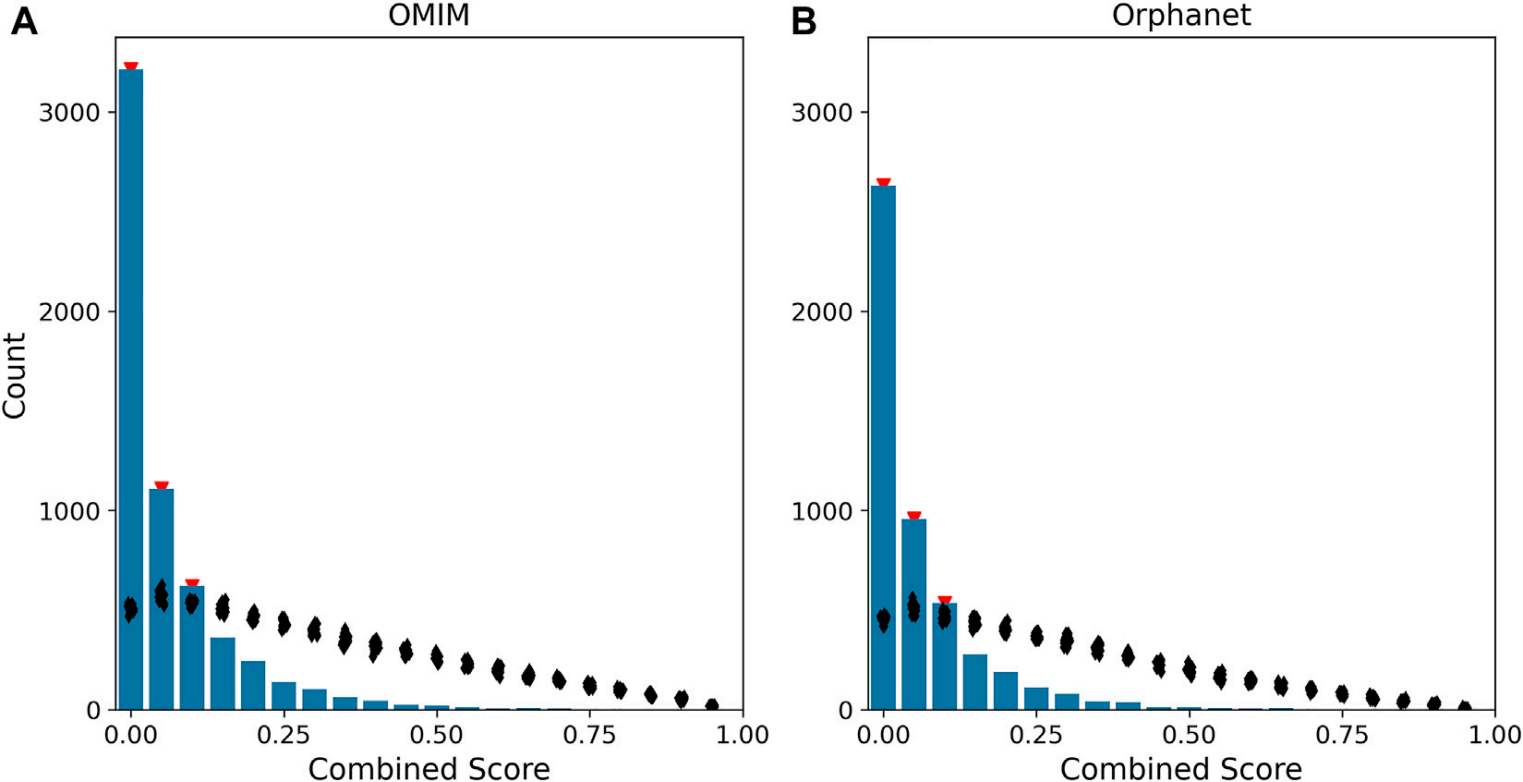
Distribution of disease scores. The histograms represent the distribution of disease scores for gene-disease associations in OMIM **(A)** and Orphanet **(B)**. The combined score of a gene is defined as the geometric mean of the rank-transformed scores of the gene for all phenotypes associated to the disease. In the figure, combined score values are binned into 20 intervals (*x*-axis). The black dots indicate the distribution of the combined score obtained after 100 randomizations of the gene-disease associations. Red triangles mark the bins having a count higher than the one obtained after randomization with *p* <0.01.

We evaluated the performance of such disease score by comparing its distribution to 100 sets of scores calculated by randomizing the gene-disease associations. The disease scores were strongly skewed to low values, with a statistically significant enrichment for scores below 0.15 (Figure 4).

In order to understand the functional implications of our scoring system, we performed enrichment analysis on the genes included in each score bin. We found that lower scores are associated to pathways related to tissue and organ development, while higher scores are weakly associated to immune-related pathways (Supplementary Figures S5, S6), indicating that our strategy is particularly well-suited to prioritize genes associated to developmental syndromes affecting multiple systems.

## 4 Discussion

We have shown that the frequency of somatic mutations observed in cancer patients is strongly associated with the involvement of a gene in genetic diseases when mutated in the germline, and is independent from other predictors such as gene age and several genomic and epigenomic features. This observation was used to build machine-learning tools to predict the causal gene of phenotypic abnormalities and genetic diseases. We believe these tools will be useful in interpreting the results of exome sequencing for genetic diseases of unknown causal genes.

Obviously, mutations causing the phenotypic abnormalities that are observed in human subjects must have a phenotypic effect on the organism but not be lethal at the cellular level. The probability that a mutation in a gene will cause an abnormal phenotype observed in an individual is positively correlated with: (1) the probability that a mutation will occur within the gene, (2) the probability that such mutation will have an effect on the phenotype; and (3) the probability that a mutation in the gene will *not* be lethal at the cellular level. Each of these is directly reflected in one of the ingredients of our model. Indeed the genomic and epigenomic factors we introduced are precisely those that are known to influence mutability (Koren et al., 2014; Monroe et al., 2022), while ancient evolutionary age can be considered as a proxy for functional relevance, and hence phenotypic effect.

The frequency of somatic mutations in cancer can be interpreted to represent the compatibility of gene mutations with cellular viability. A comprehensive study of patterns of somatic mutations in cancer (Martincorena et al., 2017) demonstrated that driver mutations, which confer an evolutionary advantage to cancer cells, represent a small minority of the somatic mutations observed. Therefore, most observed somatic mutations are presumably neutral or almost neutral in terms of cancer evolution: However, of course, only those that are viable at the cellular level will be observed. Large-scale cancer genome repositories can thus be used to assess, for each gene, the probability that a mutation will not be lethal for the cell that carries it, *i.e.* precisely the third factor described above. Compared with CRISPR-based screening approaches, such as that of (Wang et al., 2015), of gene essentiality, naturally occurring somatic mutations in tumors are more relevant to our purpose because of their *in-vivo* nature and because they assess the effect of point mutations that do not necessarily lead to complete loss of function.

Therefore, the deep relationship we found between tumor somatic mutations and genetic diseases is different and complementary to that investigated in works, such as (Kaplanis et al., 2020; Vitsios et al., 2022), which consider only driver mutations. While the relationship between *cancer-driving* mutations and genetic diseases is likely rooted in gene function, the predictive power of *total* somatic mutations is likely rooted in their ability to represent which mutations are compatible with the survival of the cell, and hence can be observed and have phenotypic effects in human subjects.

The main aim of this study was to point out that information about the frequency of somatic mutations in cancer has significant power in predicting the involvement of a gene in abnormal phenotypes. More generally, our model only uses unbiased data derived from genome-wide assays and, contrary to most disease gene prediction approaches, does not use information about gene function or frequency of germline mutations. We are not claiming our model to be a stronger predictor of IPA than those based on such data, and thus we did not compare the respective predictive performance. A natural further development is thus the integration of information about gene function and germline mutation frequency into a more comprehensive model. Another limitation of the present study is that the whole analysis was performed at the gene level: Extending it to the level of individual variants is another possible avenue for future development.

In conclusion, our results demonstrate how cancer genomics data can contribute to the task of predicting genes involved in phenotypic abnormalities, and thus potentially help in identifying disease-causing mutations in genetic disorders.
