# Supplementary material for: The frequency of somatic mutations in cancer predicts the phenotypic relevance of germline mutations
Source: Front Genet. 2023 Jan 9;13:1045301. doi: 10.3389/fgene.2022.1045301 (PMC9868957; doi:10.3389/fgene.2022.1045301)
Supplement: Supplementary file 1 [file DataSheet1.PDF]

Supplementary Material

1 SUPPLEMENTARY TABLES

Logistic Regression	
Hyperparameter Name	Hyperparameter Space
Apply PCA	Yes or No
PCA % Explained Variance	95% or 100%
L2 Regularization Strength ($C = 1/\lambda$)	0.001, 0.015, 0.215, 3.162, 46.416, 681.292, 10000
Class Weight	None or Balanced
**Random Forest**	
Hyperparameter Name	Hyperparameter Space
Apply PCA	Yes or No
PCA % Explained Variance	95% or 100%
`n_estimators`	100, 300, 1000
`max_depth`	None, 5, 10, 20
`min_samples_leaf`	1, 5, 20
`min_samples_split`	2, 5, 20
**Histogram Gradient Boost**	
Hyperparameter Name	Hyperparameter Space
Apply PCA	Yes or No
PCA % Explained Variance	95% or 100%
`max_iter`	100, 300, 500
`learning_rate`	0.01, 0.1, 1
`max_depth`	None, 5, 10, 20
`min_samples_leaf`	1, 2, 5, 10, 20
`max_leaf_nodes`	None, 31
**AdaBoost**	
Hyperparameter Name	Hyperparameter Space
Apply PCA	Yes or No
PCA % Explained Variance	95% or 100%
`n_estimators`	50, 100, 200
`learning_rate`	0.05, 1, 1.5
**Support Vector Machine**	
Hyperparameter Name	Hyperparameter Space
Apply PCA	Yes or No
PCA % Explained Variance	95% or 100%
Regularization Strength	0.001, 1, 1000
γ	0.001, 1, 1000
Class weight	Balanced or None
**Gaussian Naïve Bayes**	
Hyperparameter Name	Hyperparameter Space
Apply PCA	Yes or No
PCA % Explained Variance	95% or 100%

Table S1. Hyperparameter space explored during nested cross-validation for each candidate model.

	Mean AUROC	STD AUROC
Support Vector Machine	0.6674	0.0129
Logistic Regression	0.6680	0.0137
Random Forest	0.6877	0.0076
AdaBoost	0.6859	0.0154
Histogram Gradient Boost	0.6785	0.0162
Gaussian Naïve Bayes	0.6414	0.0096

Table S2. AUROC of all considered models, calculated using a nested cross-validation scheme with 10 outer folds and 3 inner folds.

	OMIM		Orphanet	
Rank bin	n	p-value	n	p-value
0.05	3212	0.000	2630	0.000
0.10	1108	6.280E-112	957	1.229E-112
0.15	621	1.271E-04	535	1.228E-03
0.20	362	1.000	278	1.000
0.25	243	1.000	189	1.000
0.30	140	1.000	113	1.000
0.35	102	1.000	79	1.000
0.40	63	1.000	42	1.000
0.45	45	1.000	37	1.000
0.50	24	1.000	12	1.000
0.55	20	1.000	14	1.000
0.60	12	1.000	9	1.000
0.65	6	1.000	6	1.000
0.70	7	1.000	9	1.000
0.75	6	1.000	2	1.000
0.80	0	1.000	0	1.000
0.85	2	1.000	0	1.000
0.90	1	1.000	0	1.000
0.95	0	1.000	0	1.000
1.00	1	1.000	0	9.948E-01

Table S3. Enrichment of disease scores by bin value. For genes associated to diseases in OMIM and Orphanet databases we report the number of genes in each bin of disease score (n) and the p-value of such value compared to the distribution of 100 randomizations of gene/disease associations.

2 SUPPLEMENTARY FIGURES

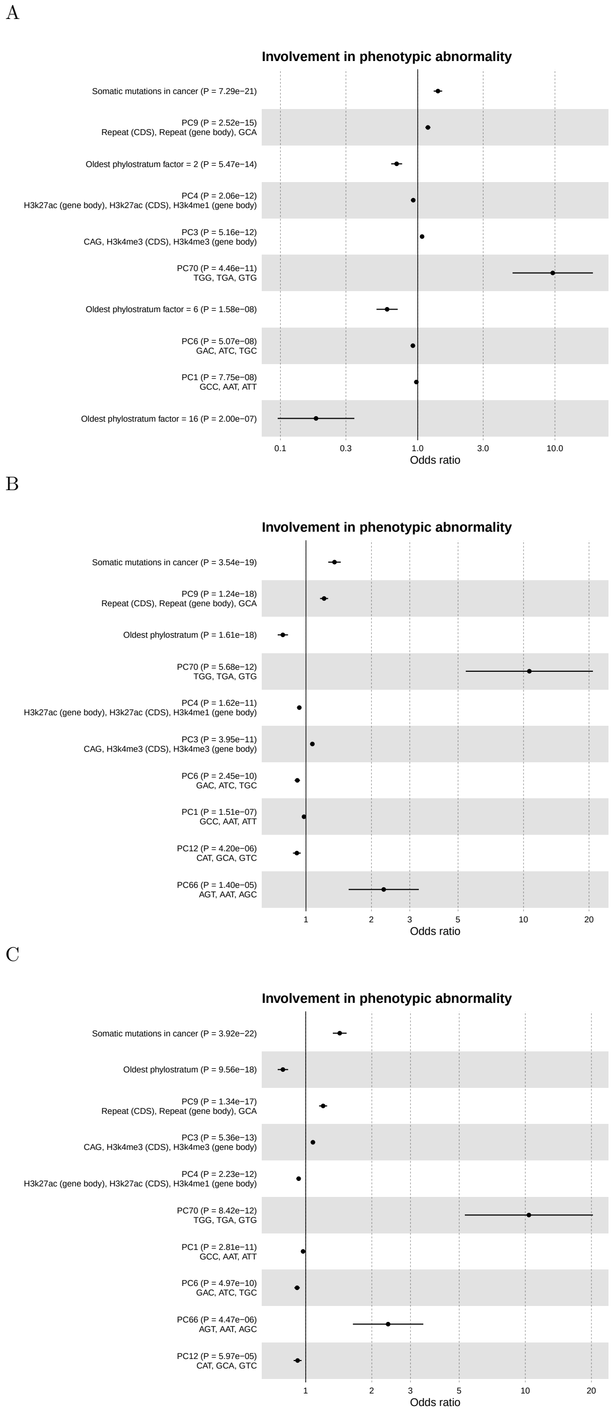

Figure S1. Multivariable logistic regression when using alternative modeling choices. (A) Gene age is treated as a categorical rather than numerical variable. (B) The rate of patients with somatic mutations in a gene is used instead of the number of patients. The rate is then averaged over tumor types. (C) Only mutations altering the protein sequence are considered.

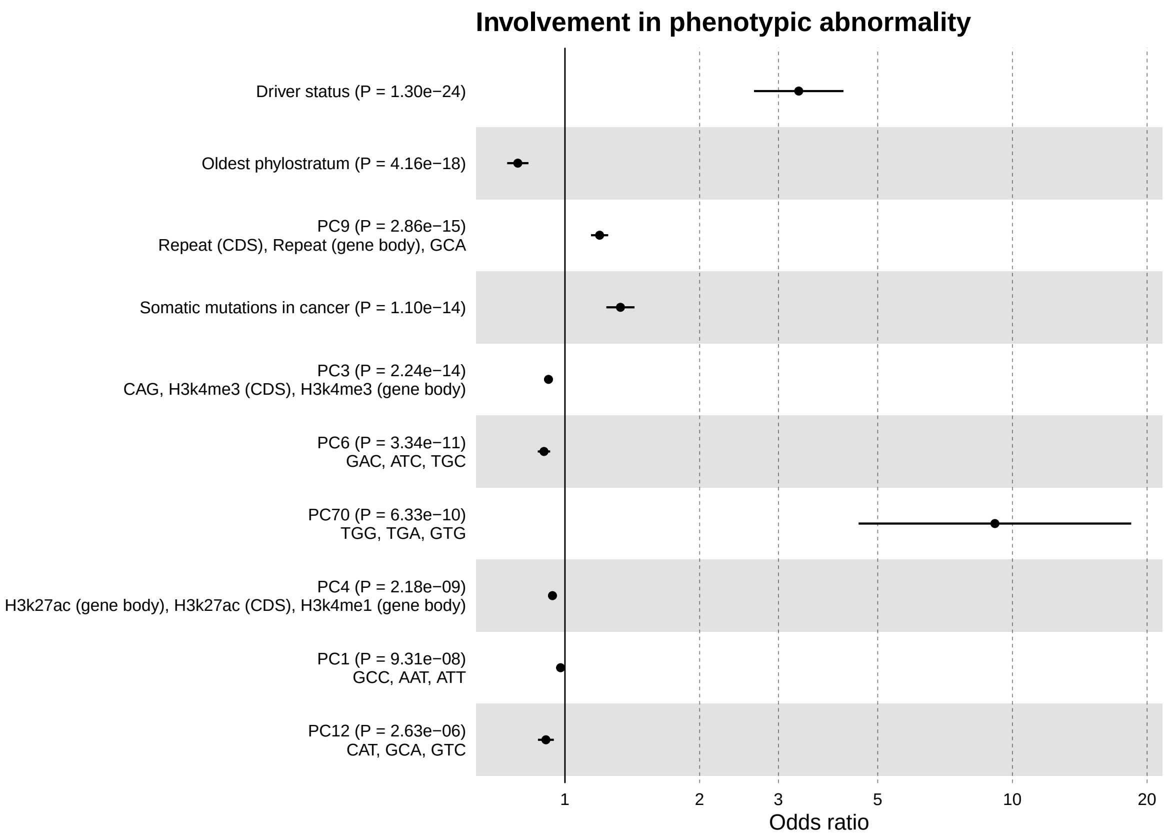

Figure S2. Multivariable logistic regression adding driver status as a further covariate. A list of 369 cancer drivers was obtained from (Martincorena et al., 2017)

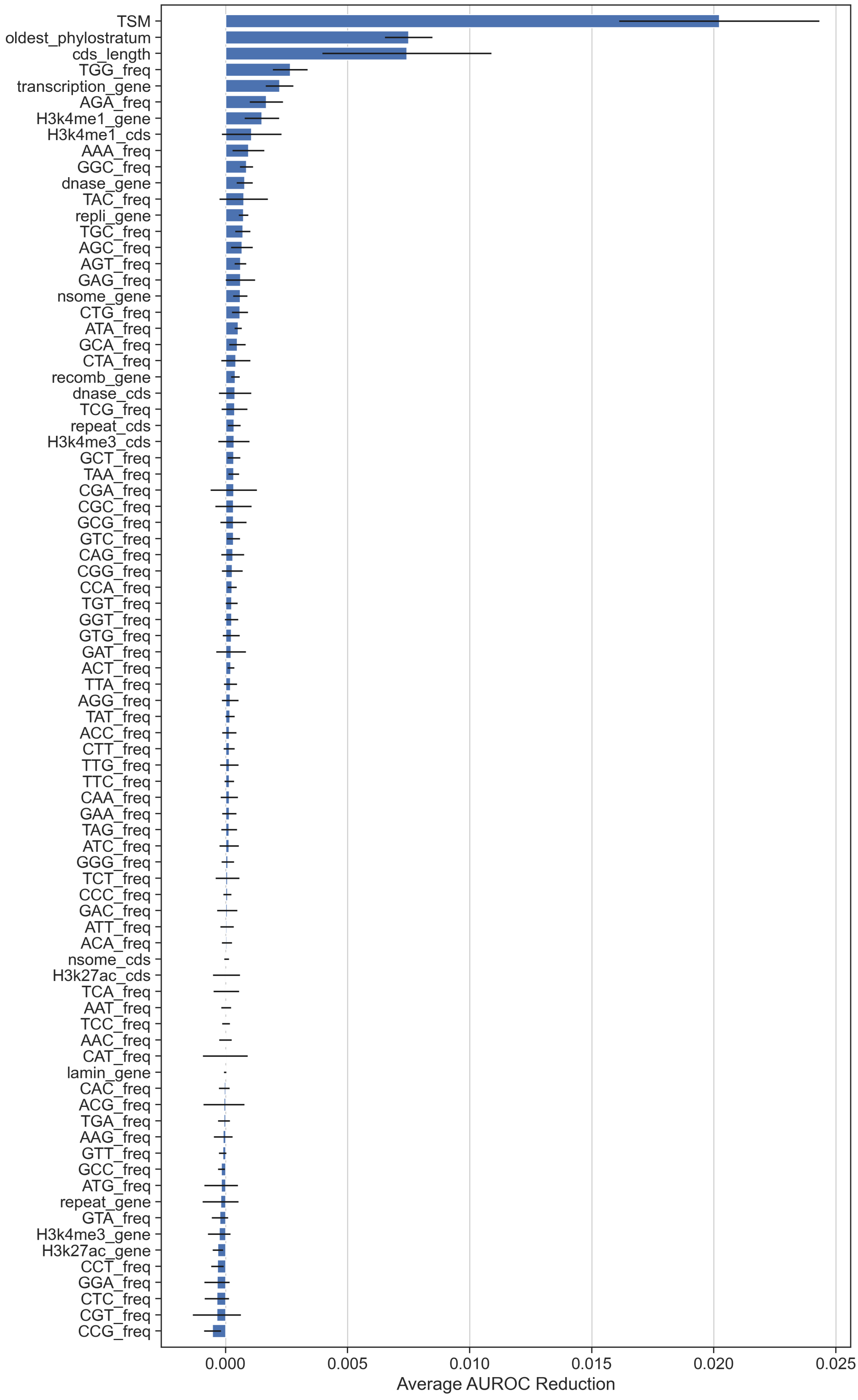

Figure S3. Feature importance scores for the Random Forest Classifier calculated by shuffling the values of each feature and measuring the loss in AUROC.

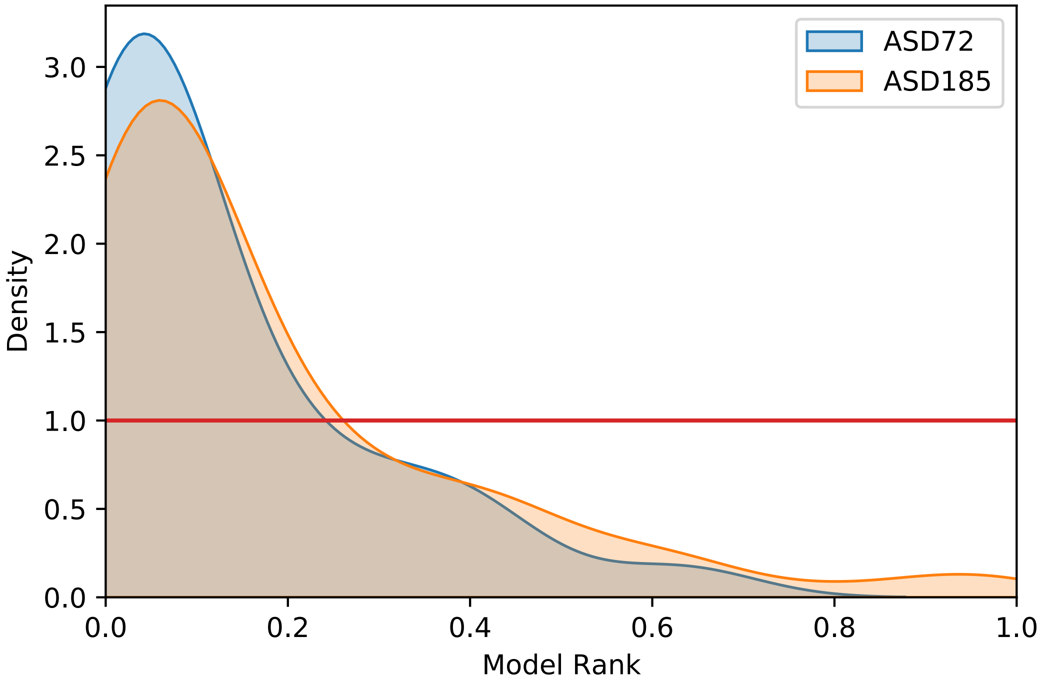

Figure S4. Rank distribution of novel genes associated to Autism Spectrum Disorder. The distribution of ranks of genes from (Fu et al., 2022) not included in our training set for ”Autism behaviour” (HP:0000729) are represented in blue (ASD72) or orange (ASD185). The uniform distribution of the ranks of all genes is represented in red. Both distributions were significantly different from the uniform distribution ($U = 61033$, $P = 1.173 \cdot 10^{-12}$ for ASD72 and $U = 342195$, $P = 1.261 \cdot 10^{-34}$ for ASD185, Mann-Whitney test).

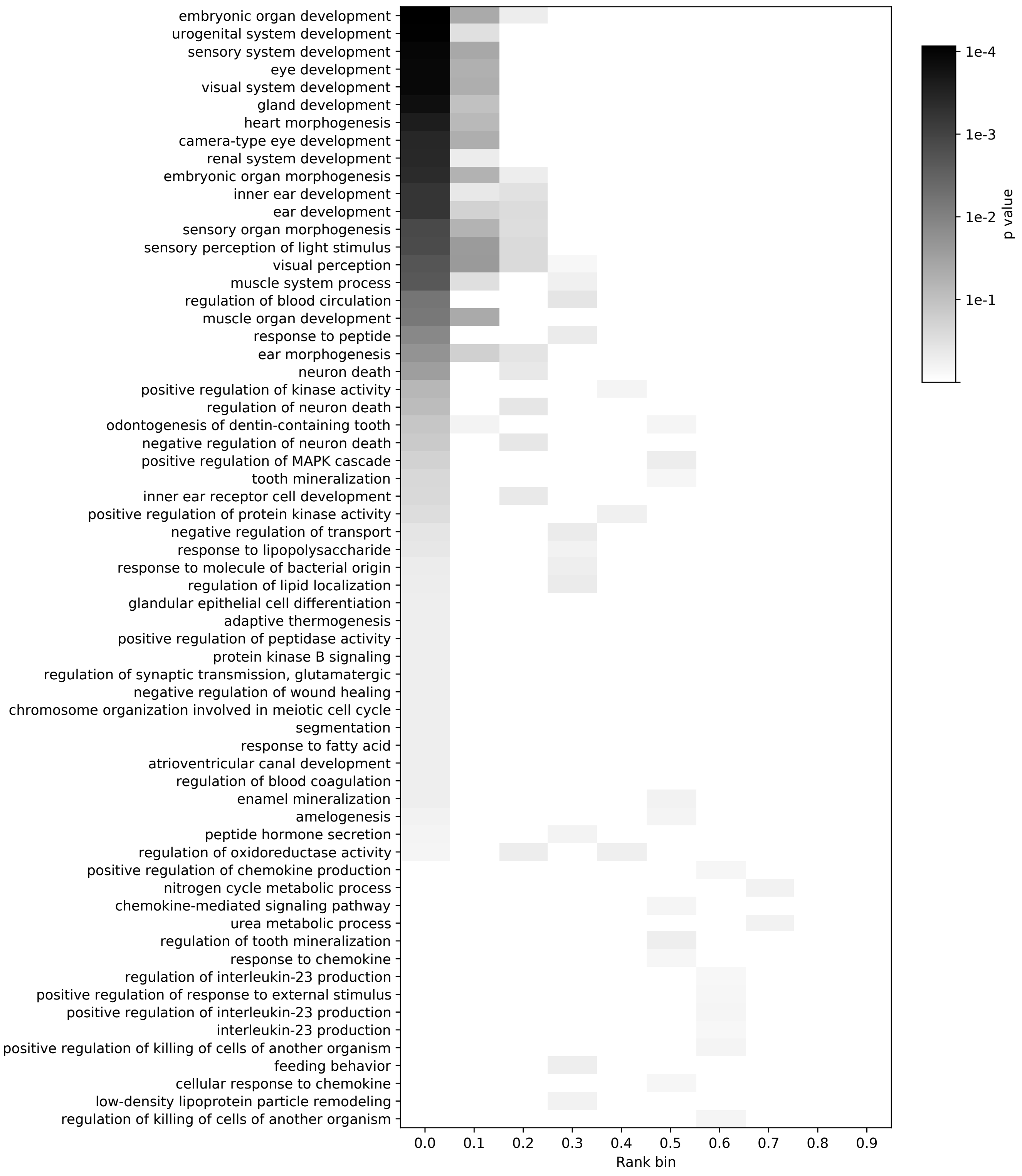

Figure S5. Gene Ontology term enrichment analysis of genes associated to OMIM diseases, classified by their disease score. Each column represent a bin of compound score, each row a GO:BP term that has been found enriched at least once. Darker cells are indicative of lower P-values.

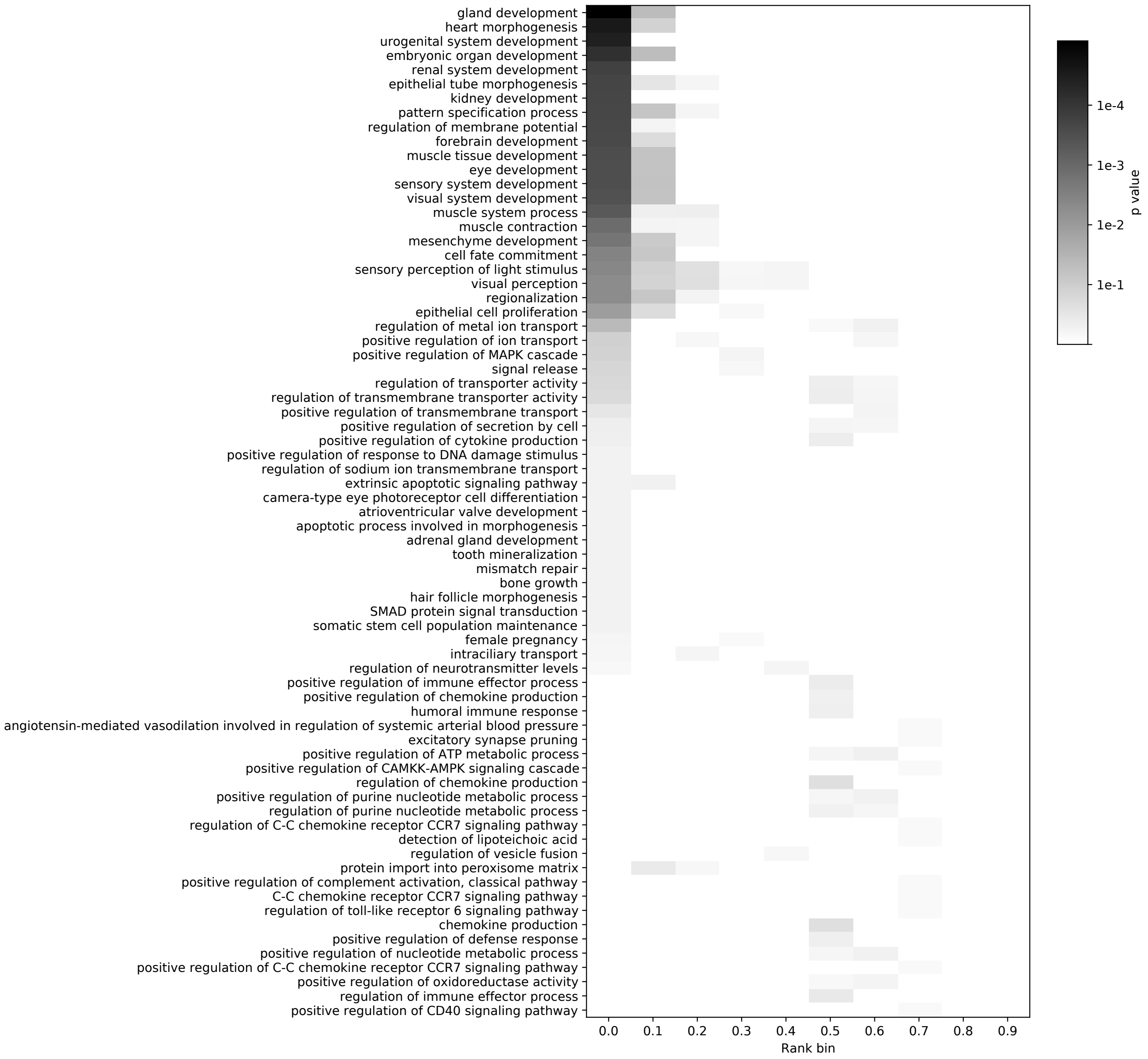

Figure S6. Gene Ontology term enrichment analysis of genes associated to Orphanet diseases, classified by their disease score. Each column represent a bin of compound score, each row a GO:BP term that has been found enriched at least once. Darker cells are indicative of lower P-values.

REFERENCES

Fu, J. M., Satterstrom, F. K., Peng, M., Brand, H., Collins, R. L., Dong, S., et al. (2022). Rare coding variation provides insight into the genetic architecture and phenotypic context of autism. *Nat Genet* doi:10.1038/s41588-022-01104-0

Martincorena, I., Raine, K. M., Gerstung, M., Dawson, K. J., Haase, K., Van Loo, P., et al. (2017). Universal Patterns of Selection in Cancer and Somatic Tissues. *Cell* 171, 1029–1041.e21. doi:10.1016/j.cell.2017.09.042
